# Supplementary material for: Haplotype in SERPINA1 (AAT) Is Associated with Reduced Risk for COPD in a Mexican Mestizo Population
Source: Int J Mol Sci. 2019 Dec 27;21(1):195. doi: 10.3390/ijms21010195 (PMC6982076; doi:10.3390/ijms21010195)
Supplement: Supplementary file 1 [file ijms-21-00195-s001.pdf]

Supplementary tables

Table S1. Demographic comparison between COPD-S *vs.* COPD-BB groups.

Variable	COPD-S (n=297)	COPD-BB (n=178)	*p*-value
Age, years	67 (50-80)	74 (51-86)	<0.001
Male (%)	76.35 (%)	8 (%)	<0.001
Body mass index	24.5 (13.5-36.1)	24.5 (18.7-32.7)	0.098
**GOLD**			
GOLD I	14.73 [%]	27.21 [%]	0.002
GOLD II	44.52 [%]	54.41 [%]	0.057
GOLD III	29.11 [%]	15.44 [%]	0.002
GOLD IV	11.64 [%]	2.94 [%]	0.003
**Spirometry**			
FVC (%) post	78 (24-155)	79 (43-144)	0.234
FEV1 (%) post	42 (12-88)	48 (18-94)	0.002
FEV1/FVC (%) post	52 (26.1-69.4)	60.9 (36.7-68.4)	<0.001

COPD-S = Patients with COPD related to tobacco smoking, COPD-BB = Patients with COPD related to biomass-burning. All values are shown in median and values in brackets are shown as the minimum and maximum values. Measures of lung function post-bronchodilator use. *p*-value <0.05 was significative. We used median test to make comparisons between groups.

Table S2. Demographic comparison between FE-S *vs.* FE-BB groups.

Variable	FE-S (n=32)	FE-BB (n=38)	*p*-value
Age, years	67 (52-77)	68 (61-86)	0.230
Male (%)	78.12 (%)	10.53 (%)	<0.001
Body mass index	24.6 (15.2-35.9)	23.8 (20-30.3)	0.224
**GOLD**			
G I (I – II), %	14 [43.75]	22 [81.48]	0.039
G II (III – IV), %	18 [56.25]	5 [18.52]	
**Spirometry**			
FVC (%) post	76.5 (32-121)	87 (69-135)	0.043
FEV1 (%) post	35.5 (14-66)	51 (34-60)	0.011
FEV1/FVC (%) post	48.4 (29.1-65.8)	55.6 (37-69)	0.116

FE-S = Patients with COPD related to tobacco smoking frequent exacerbators, FE-BB = Patients with COPD related to biomass burning frequent exacerbators. G1 = GOLD I and GOLD II stages, G2 = GOLD III and GOLD IV stages. All values are shown in median and values in brackets are shown as minimum and maximum. Measures of lung function post-bronchodilator use. *p*-value <0.05 was significative. We used median test to make comparisons between groups.

Table S3. Logistic regression analysis by co-variables in the COPD-S group.

Tobacco smoking comparison

CHR	SNP	BP	A1	TEST	NMISS	OR	SE	L95	U95	STAT	P
14	rs1303	94378506	3	ADD	914	1.077	0.1448	0.8113	1.431	0.5153	0.6063
14	rs1303	94378506	3	DOMDEV	914	1.023	0.1971	0.695	1.505	0.1146	0.9088
14	rs1303	94378506	3	COV1	914	1.124	0.01089	1.101	1.149	10.75	5.82E-27
14	rs1303	94378506	3	COV2	914	1.033	0.00418	1.025	1.042	7.777	7.44E-15
14	rs1303	94378506	3	GENO_2DF	914	NA	NA	NA	NA	0.4149	0.8127
14	rs709932	94382864	4	ADD	914	0.8856	0.3594	0.4378	1.791	-0.3381	0.7353
14	rs709932	94382864	4	DOMDEV	914	1.489	0.4077	0.6696	3.31	0.9763	0.3289
14	rs709932	94382864	4	COV1	914	1.124	0.01088	1.101	1.149	10.78	4.16E-27
14	rs709932	94382864	4	COV2	914	1.033	0.004201	1.025	1.042	7.728	1.09E-14
14	rs709932	94382864	4	GENO_2DF	914	NA	NA	NA	NA	1.81	0.4046

cov1 Age

cov2 Tobacco index

Table S4. Logistic regression analysis by co-variables in the COPD-BB group.

Biomass-burning comparison

CHR	SNP	BP	A1	TEST	NMISS	OR	SE	L95	U95	STAT	P
14	rs1303	94378506	3	ADD	657	0.8561	0.1515	0.6361	1.152	-1.026	0.3051
14	rs1303	94378506	3	DOMDEV	657	0.7758	0.2203	0.5037	1.195	-1.152	0.2492
14	rs1303	94378506	3	COV1	657	1.077	0.01005	1.056	1.099	7.412	1.25E-13
14	rs1303	94378506	3	COV2	657	1.002	0.000514	1.001	1.003	3.593	0.0003274
14	rs1303	94378506	3	GENO_2DF	657	NA	NA	NA	NA	3.471	0.1763
14	rs709932	94382864	4	ADD	657	5.45E-05	4776	0	inf	-0.002055	0.9984
14	rs709932	94382864	4	DOMDEV	657	1.54E+04	4776	0	inf	0.002019	0.9984
14	rs709932	94382864	4	COV1	657	1.076	0.01003	1.055	1.098	7.334	2.24E-13
14	rs709932	94382864	4	COV2	657	1.002	0.000517	1.001	1.003	3.559	0.0003729
14	rs709932	94382864	4	GENO_2DF	657	NA	NA	NA	NA	0.2962	0.8624

cov1 Age

cov2 Biomass exposure index

Supplementary Figures

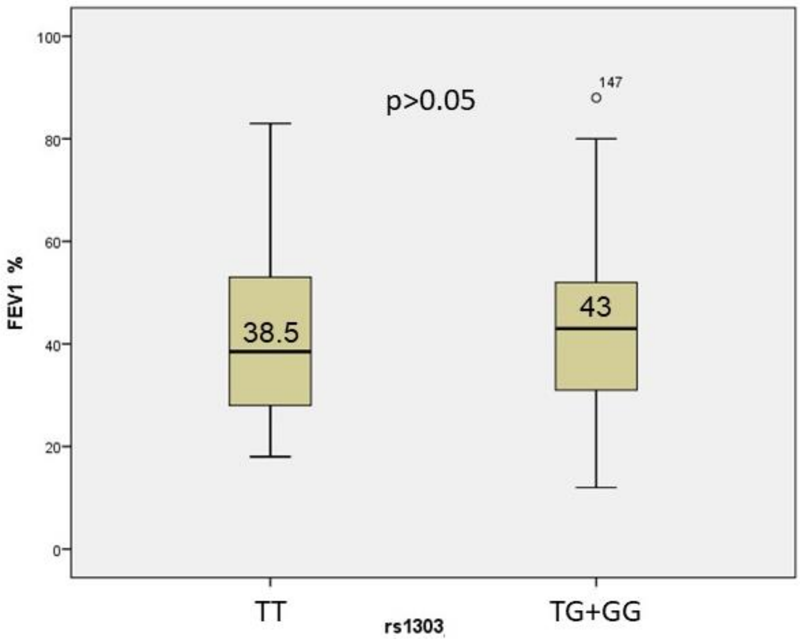

Figure S1. Median FEV1(%) based on the genotypes obtained for rs1303 in the COPD-S group. We used the Median test to compare groups.

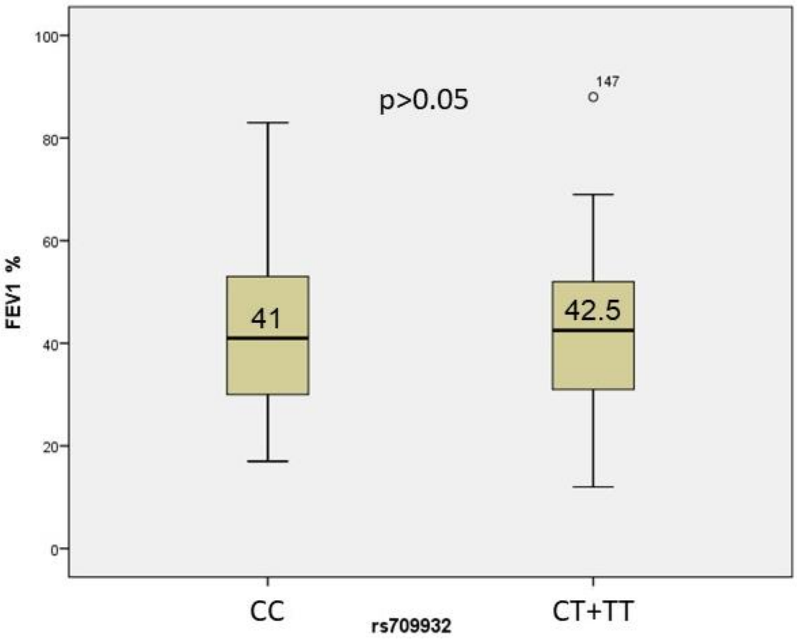

Figure S2. Median FEV1(%) based on the genotypes obtained for rs709932 in the COPD-S group. We used the Median test to compare groups.

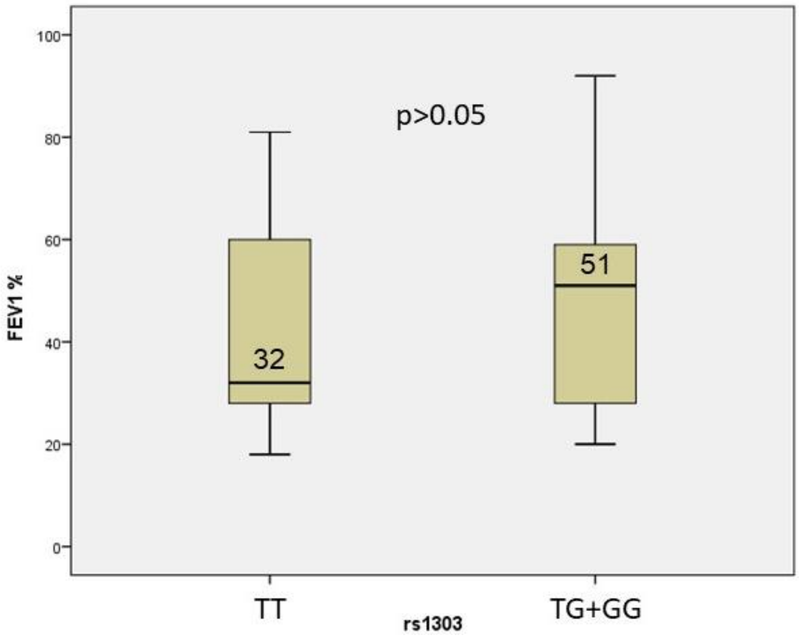

Figure S3. Median FEV1(%) based on the genotypes obtained for rs1303 in the COPD-BB group. We used the Median test to compare groups.

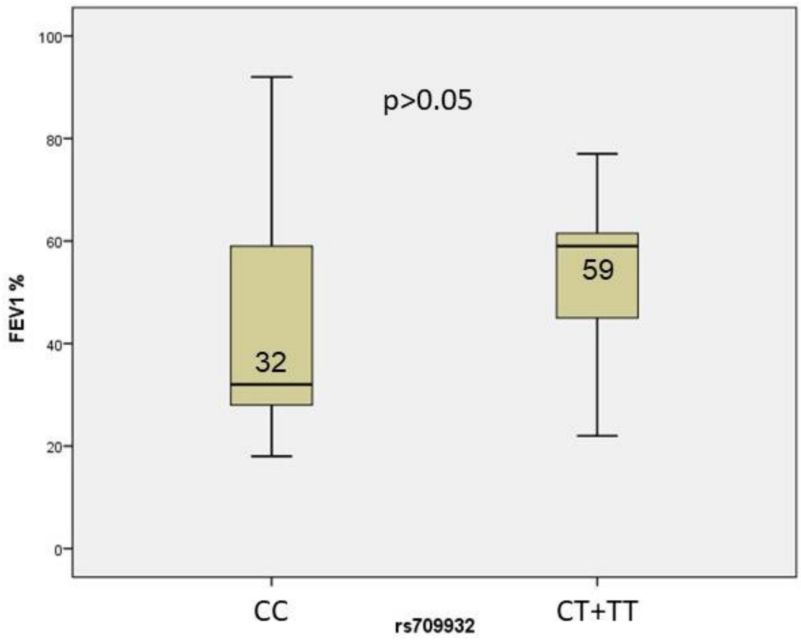

Figure S4. Median FEV1(%) based on the genotypes obtained for rs709932 in the COPD-BB group. We used the Median test to compare groups.

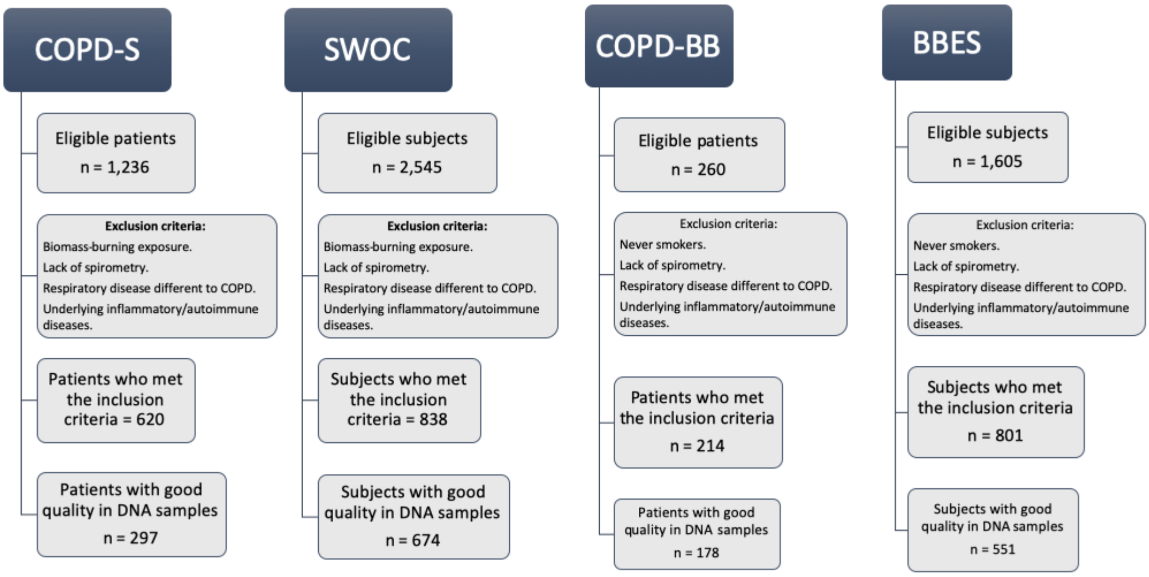

Figure S5. Flowchart with enrolled patients.
